# Quantitative Assessment of the Association between Genetic Variants in MicroRNAs and Colorectal Cancer Risk

**DOI:** 10.1155/2015/276410

**Published:** 2015-05-20

**Authors:** Xiao-Xu Liu, Meng Wang, Dan Xu, Jian-Hai Yang, Hua-Feng Kang, Xi-Jing Wang, Shuai Lin, Peng-Tao Yang, Xing-Han Liu, Zhi-Jun Dai

**Affiliations:** ^1^Department of Oncology, The Second Affiliated Hospital of Xi'an Jiaotong University, Xi'an 710004, China; ^2^Center for Translational Medicine, Frontier Institute of Science and Technology (FIST), Xi'an Jiaotong University, Xi'an 710049, China; ^3^Department of Chemistry, School of Science, Xi'an Jiaotong University, Xi'an 710049, China

## Abstract

*Background*. The associations between polymorphisms in microRNAs and the susceptibility of colorectal cancer (CRC) were inconsistent in previous studies. This study aims to quantify the strength of the correlation between the four common polymorphisms among microRNAs (hsa-mir-146a rs2910164, hsa-mir-149 rs2292832, hsa-mir-196a2 rs11614913, and hsa-mir-499 rs3746444) and CRC risk. *Methods*. We searched PubMed, Web of Knowledge, and CNKI to find relevant studies. The combined odds ratio (OR) with 95% confidence interval (95% CI) was used to estimate the strength of the association in a fixed or random effect model. *Results*. 15 studies involving 5,486 CRC patients and 7,184 controls were included. Meta-analyses showed that rs3746444 had association with CRC risk in Caucasians (OR = 0.57, 95% CI = 0.34–0.95). In the subgroup analysis, we found significant associations between rs2910164 and CRC in hospital based studies (OR = 1.24, 95% CI = 1.03–1.49). rs2292832 may be a high risk factor of CRC in population based studied (OR = 1.18, 95% CI = 1.08–1.38). *Conclusion*. This meta-analysis showed that rs2910164 and rs2292832 may increase the risk of CRC. However, rs11614913 polymorphism may reduce the risk of CRC. rs3746444 may have a decreased risk to CRC in Caucasians.

## 1. Introduction

Colorectal cancer (CRC) is the third leading cause of cancer death for both men and women in the USA [1]. In Europe, CRC represents one of the primary causes of cancer deaths [2], and, in Asia, CRC is the fourth leading cause of mortality by cancer, and its incidence is increasing [3]. Epidemiological evidences have suggested that the risk of colorectal neoplasm is affected by multiple factors including a positive family history, excessive meat consumption, smoking status, and alcohol consumption, as well as genetic alterations, such as genetic polymorphisms [2].

However, the mechanism of colorectal carcinogenesis is still not fully understood. Compared with other complex diseases, CRC may be caused by both genetic and environmental factors [4]. Because well-recognized genetic predisposition syndromes account for less than 3% of CRC, low-penetrance genetic factors alone or in combination with environmental factors probably contribute to the development of CRC [5].

MicroRNAs (miRNAs) are a large class of small noncoding RNAs, which participate in various biological processes and may regulate tumor suppressor genes or oncogenes [6]. Single nucleotide polymorphisms (SNPs) in miRNAs may alter the expression, processing, and transcription of miRNAs and thus contribute to cancer development [7]. Recently, many epidemiological studies have demonstrated that some SNPs in the miRNA genes could alter miRNA expression and/or maturation and also be associated with the susceptibility and progression of cancer [8]. Rs2910164 in hsa-mir-146a locus resides at position +60 relative to the first nucleotide of the pre-miR-146a gene. The previous studies have shown that miR-146a plays an important role in cell proliferation and metastatic ability in cancers and the variation of miR-146a may be involved in carcinogenesis [9, 10]. MiR-196a2 includes two different mature miRNAs (miR-196a-5P and miR-196a-3P) and rs11614913 located in the mature sequence of miR-196a-3P. Rs11614913 could influence the expression levels of mature miR-196a and may have an impact on the expression of its target gene, which may play a role in regulating carcinogenesis [10]. It has been identified that rs3746444 is located in the stem region of the mir-499 gene. And several studies have identified miR-499 rs3746444 as a possible biomarker for some cancers [11–13]. Compared with other three genes, there were few studies of rs2292932 in miR-149 up to now. But the T allele in miR-149 might be masked by the presence of other unidentified causal genes involved in cancer development [14]. Furthermore, several Genome-wide association studies have reported that these four common SNPs may be associated with cancer risk [8]. However, results of these studies remain inconsistent. To provide a more precise estimate of the association of miRNA polymorphisms with CRC risk, we performed a meta-analysis on all eligible published case-control studies and evaluated the effect of the four SNPs on CRC risk.

## 2. Methods

### 2.1. Publication Search

Relevant articles were independently searched by two authors (Wang M and Dai ZJ) in PubMed, Web of Science, and Chinese National Knowledge Infrastructure (CNKI). The keywords were as follows: colorectal cancer/colorectal carcinoma, microRNA 146a/149/196a2/499, and polymorphism/genotype/variant/SNP. All qualified studies prior to December 20, 2014, were included. The eligible literature must be published in English or Chinese. Furthermore, reference lists of main reports and review articles were also reviewed manually to identify additional relevant publications.

### 2.2. Selection Criteria

The following criteria were used to select eligible studies for further meta-analysis: (1) case-control design; (2) full-text study; (3) studies that evaluated the associations between miRNA polymorphisms and CRC risk; and (4) studies that included detailed genotyping data.

### 2.3. Data Extraction

Articles were reviewed independently by two reviewers and data with discrepancies were discussed by all authors. For each included study, the following information was collected: first author, year of publication, country of origin, ethnicity, source of control, total number of cases and controls, and genotyping methods as well as number of cases and controls with the different genotypes. Different ethnic groups were categorized as Caucasian, Asian, African, and “mixed.” All the case and control groups were well controlled.

### 2.4. Statistical Analysis

The associations between miRNA polymorphisms and CRC risk were measured by odds ratio (OR) with 95% confidence interval (CI). The significance of the pooled OR was determined by the *Z* test.

The meta-analysis assessed association by using 5 different genetic models: homozygous genetic model, heterozygote genetic model, dominant genetic model, recessive genetic model, and allelic model. Hardy-Weinberg equilibrium (HWE) among the control subjects was tested by the Chi-square test. Statistical heterogeneity among studies was assessed with the *Q* and *I* ^2^ statistics. If the *P* value of heterogeneity test was more than 0.05 (*P* > 0.05), the pooled OR estimate of the study was calculated by the fixed-effects model. Otherwise, the random-effects model was used. The value of the *I* index was used to assess the degree of heterogeneity (*I* ^2^ < 25%: no heterogeneity; 25% < *I* ^2^ < 50%: moderate heterogeneity; 50% < *I* ^2^ < 75%: high heterogeneity; and *I* ^2^ > 75%: extreme high heterogeneity). Publication bias was evaluated by the funnel plot. All statistical analyses were carried out with the review manager version 5.1 (Revman; The Cochrane Collaboration, Oxford, UK).

## 3. Results

### 3.1. Characteristics of Studies

**Figure 1:**
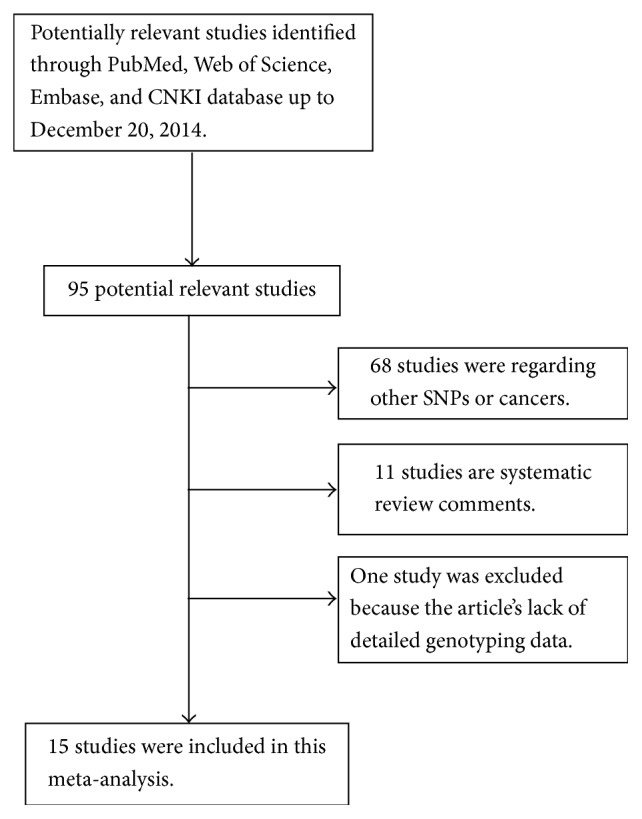
Flow chart of study selection.

According to the inclusion criteria defined above (Figure 1), fifteen studies on miRNA polymorphisms with CRC risk were identified [8], including 5,486 CRC patients and 7,184 cancer-free controls. All the included eligible studies were published in English or Chinese. Among the eligible fifteen studies, eleven studies were based on Asian backgrounds which were carried out in China and Korea. Only four studies were based on Caucasian, having been carried out in Greek, Lithuania, Italy, and Czech, respectively. Simultaneously, there were seven hospital based studies and eight population based studies. Main characteristics of the included studies were listed in Table 1.

### 3.2. Meta-Analysis Results

As shown in Table 2, the frequencies of the minor allele varied widely across the eligible studies, ranging from 0.14 to 0.80 (rs2910164), 0.33 to 0.82 (rs2292832), 0.34 to 0.66 (rs11614913), and 0.14 to 0.68 (rs3746444). The average frequencies of the minor allele in the four polymorphisms were 0.47, 0.66, 0.51, and 0.27, respectively. The distributions of genotypes in the controls were all in agreement with HWE except four studies [8, 15–17].

**Figure 2:**
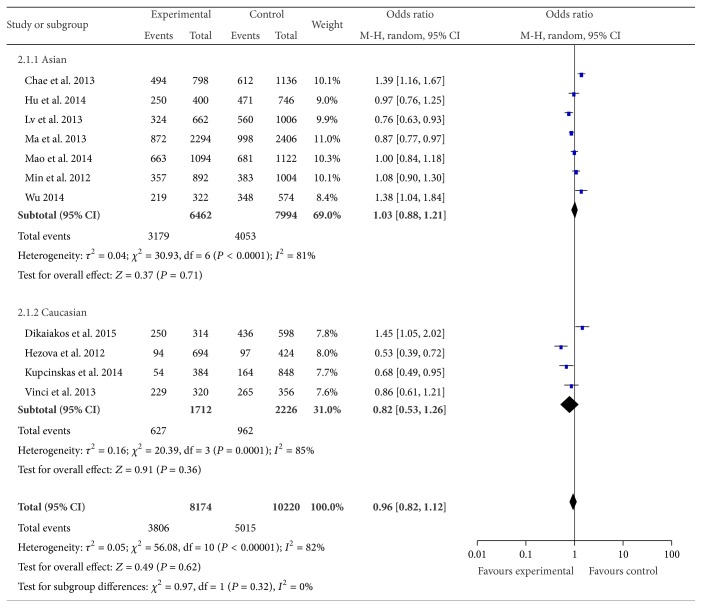
Forest plot of miR-146a rs2910164 polymorphism and CRC risk (C versus G). The squares and horizontal lines correspond to the study specific OR and 95% CI. The area of the squares reflects the weight (inverse of the variance). The diamond represents the summary OR and 95% CI.

The main results of this meta-analysis were presented in Table S1, in Supplementary Material available online at http://dx.doi.org/10.1155/2015/276410. There were 11 studies with 3,937 cases and 5,120 controls for rs2910164. miR-146a rs2910164 polymorphism has no association with CRC risk in the overall population (C versus G: OR = 0.96, 95% CI = 0.82–1.12, *P* = 0.62, Figure 2; CC versus GG: OR = 1.02, 95% CI = 0.78–1.33, *P* = 0.89; GC versus GG: OR = 1.00, 95% CI = 0.83–1.20, *P* = 0.97; CC versus GG + GC: OR = 1.03, 95% CI = 0.80–1.31, *P* = 0.83; GC + CC versus GG: OR = 1.01, 95% CI = 0.84–1.22, *P* = 0.89). After omitting the study which was not according with the HWE, the results were in accordance with the overall population (C versus G: OR = 0.93, 95% CI = 0.79–1.09, *P* = 0.38; CC versus GG: OR = 0.97, 95% CI = 0.73–1.28, *P* = 0.83; GC versus GG: OR = 0.98, 95% CI = 0.81–1.20, *P* = 0.88; CC versus GG + GC: OR = 0.99, 95% CI = 0.76–1.27, *P* = 0.91; GC + CC versus GG: OR = 0.99, 95% CI = 0.82–1.20, *P* = 0.90). When stratifying analysis by ethnicity, there was also no significant association observed between the rs2910164 and CRC susceptibility in the five genetic models. In the analysis stratified by the source of control, significant associations were observed in the hospital based studies for recessive genetic model (CC versus GG + GC: OR = 1.24, 95% CI = 1.03–1.49, *P* = 0.02). No associations were found in population based studies.

**Figure 3:**
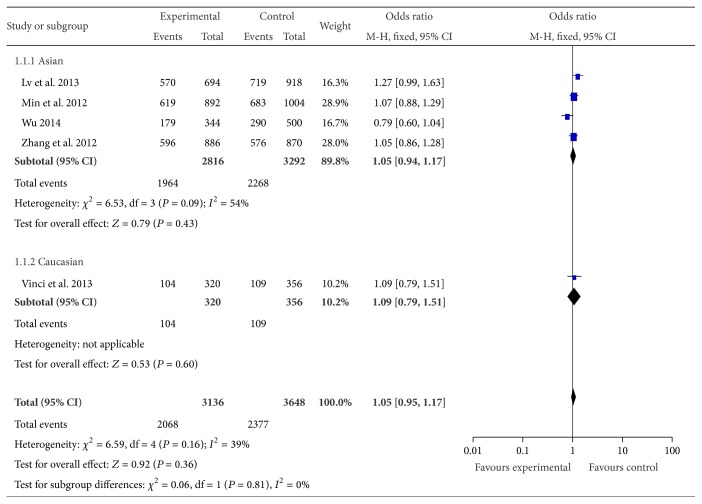
Forest plot of miR-149 rs2292832 polymorphism and CRC risk (T versus C). The squares and horizontal lines correspond to the study specific OR and 95% CI. The area of the squares reflects the weight (inverse of the variance). The diamond represents the summary OR and 95% CI.

The association of the rs2292832 polymorphism with CRC susceptibility was investigated in 5 studies with 1,568 cases and 1,824 controls. We failed to find any significant associations in any genotype (T versus C: OR = 1.05, 95% CI = 0.95–1.17, *P* = 0.36, Figure 3; TT versus CC: OR = 1.09 95% CI = 0.87–1.37, *P* = 0.44; CT versus CC: OR = 1.35, 95% CI = 0.64–2.86, *P* = 0.43; TT versus CC + CT: OR = 0.91, 95% CI = 0.54–1.53, *P* = 0.71; TT + CT versus CC: OR = 1.24, 95% CI = 0.82–1.87, *P* = 0.31). And there was also no association existing in any genetic models after we rejected the studies which were not in agreement with HWE. There was only one study based on Caucasian. When excluding the Caucasian study, the null association remained in Asians. We further made stratified analysis based on the source of control. And we observed significant associations in population based studies in the recessive genetic model (TT versus CC + CT: OR = 1.18, 95% CI = 1.08–1.38, *P* = 0.04).

**Figure 4:**
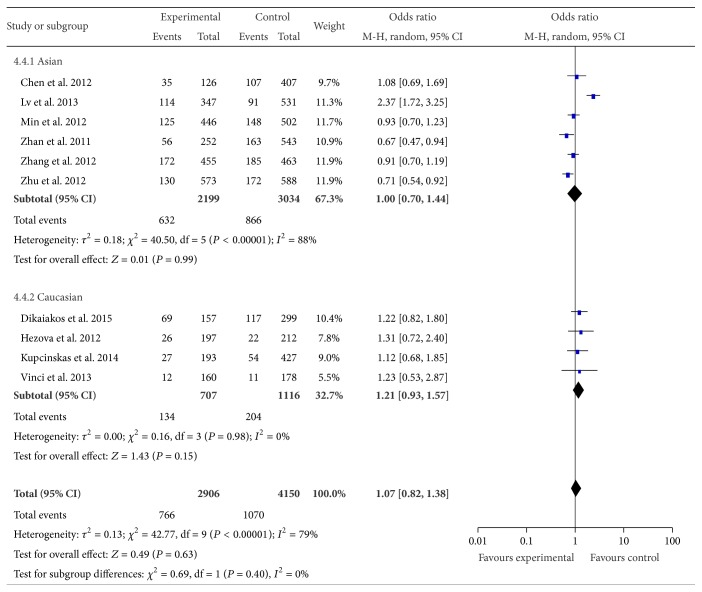
Forest plot of miR-196a2 rs11614913 polymorphism and CRC risk (TT versus CC + CT). The squares and horizontal lines correspond to the study specific OR and 95% CI. The area of the squares reflects the weight (inverse of the variance). The diamond represents the summary OR and 95% CI.

Ten studies with 2,906 cases and 4,150 controls were used to evaluate the relationship between rs11614913 polymorphism and CRC risk. No significant association was detected under all the genetic models (haploid model: OR = 1.10, 95% CI = 0.84–1.43, *P* = 0.50; homozygote comparison: OR = 1.18, 95% CI = 0.76–1.83, *P* = 0.47; heterozygote comparison: OR = 1.11, 95% CI = 0.82–1.50, *P* = 0.49; dominant model: OR = 1.13, 95% CI = 0.83–1.55, *P* = 0.44 and recessive model: OR = 1.07, 95% CI = 0.82–1.38, *P* = 0.63, Figure 4). When excluding the studies which were inconsistent with HWE, the results showed a significant association between rs11614913 polymorphism and CRC risk in the homozygous genetic model (TT versus CC: OR = 0.80, 95% CI = 0.68–0.95, *P* = 0.009). Further subgroup analysis by ethnicity showed no association between rs11614913 and CRC risk either in Caucasians or in Asians. And no significant associations were observed in population based studies and hospital based studies for all genetic models.

**Figure 5:**
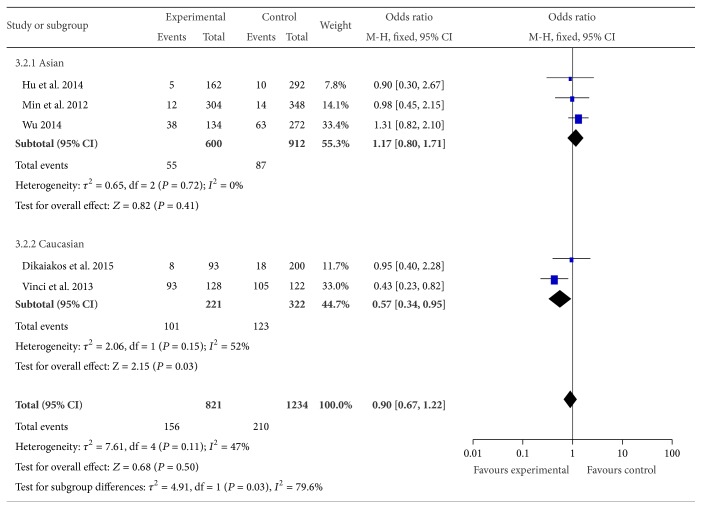
Forest plot of miR-499 rs3746444 polymorphism and CRC risk (GG versus AA). The squares and horizontal lines correspond to the study specific OR and 95% CI. The area of the squares reflects the weight (inverse of the variance). The diamond represents the summary OR and 95% CI.

For miR-499 rs3746444 polymorphism, our meta-analysis contained 6 studies with 1,471 cases and 2,104 controls. Overall, the rs3746444 polymorphism has no association with CRC risk (haploid model: OR = 0.96, 95% CI = 0.84–1.10, *P* = 0.58; homozygote comparison: OR = 0.90, 95% CI = 0.67–1.22, *P* = 0.50; heterozygote comparison: OR = 0.83, 95% CI = 0.54–1.26, *P* = 0.38; dominant model: OR = 0.91, 95% CI = 0.71–1.17, *P* = 0.45 and recessive model: OR = 0.95, 95% CI = 0.73–1.25, *P* = 0.73). Further analysis of the studies which were in agreement with HWE also showed no association between the rs3746444 polymorphism and CRC. And null associations were found in hospital based studies or population based studies. However, in the stratified analysis according to ethnicity, the homozygote model demonstrated a significant decrease in the CRC risk in Caucasians (GG versus AA: OR = 0.57, 95% CI = 0.34–0.95, *P* = 0.03, Figure 5).

### 3.3. Publication Bias

**Figure 6:**
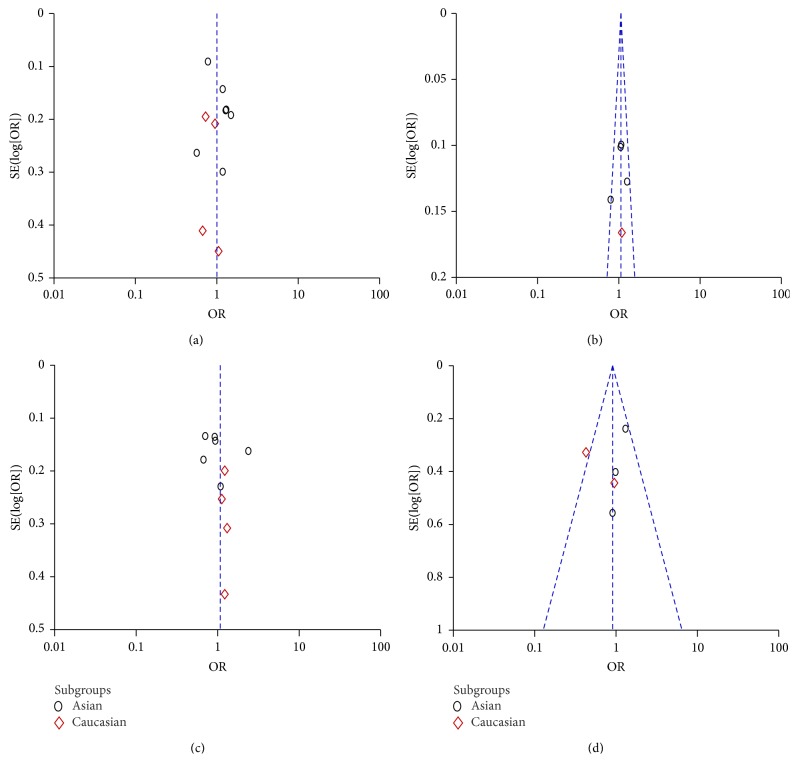
Funnel plot assessing evidence of publication bias from the eligible studies. (a) miR-146a rs2910164; (b) miR-149 rs2292832; (c) miR-196a2 rs11614913; (d) miR-499 rs3746444.

In this meta-analysis, we performed funnel plot to access the publication bias. As showed in Figure 6, the funnel plots failed to reveal any obvious asymmetry in all genotypes in overall population. Therefore, the results indicated that publication bias was not significant in this meta-analysis.

## 4. Discussion

Allelic variants in the sequence of mature miRNAs represent a particularly interesting potential source of phenotypic diversity of genetic diseases, which may contribute directly to disease susceptibility [18]. Accumulating evidence has shown that miRNAs regulate the expression of roughly 30% of the all human genes through posttranscriptional mechanisms [19]. SNPs in miRNA genes could function through three ways: firstly, the transcription of the primary transcript; secondly, pri-miRNA and pre-miRNA processing; and thirdly, effects on miRNA-mRNA interactions [7]. Genetic effects connected to SNPs at the level of miRNA genes may have a significant relationship with the expression and clinical features in CRC [16].

A large number of studies had highlighted several associations between SNPs in miRNAs and the risk of CRC [8]. However, controversial experimental data were obtained. For example, there was no significant association between rs2910164 and CRC in Hezova et al.'s study [20], whereas Chae et al. reported that miR-146a rs2910164 polymorphism of genotype CC may contribute to a higher risk of CRC [21]. Min et al. from Korea [22] demonstrated that miR-196a2 rs11614913 polymorphism had a decreased risk to CRC, whereas Lv et al. [8] reported that T allele in rs11614913 was associated with an increased risk of CRC compared with the C allele.

The present meta-analysis, including 5,486 CRC patients and 7,184 cancer-free controls from 15 case-control studies, was conducted to evaluate the association between the four common SNPs in miRNAs (miR-146a rs2910164, miR-149 rs2292832, miR-196a2 rs11614913, and miR-499 rs3746444) and CRC risk. In this study, we found that rs3746444 polymorphism has no association with CRC risk in the overall population. However, accumulative data from two studies based on Caucasian background (317 cases and 477 controls) showed significantly decreased cancer risks in the homozygous model. Considering the limited sample size, we need more large well-designed studies to evaluate the result. Simultaneously, we failed to find any significant correlation between the rs2910164, rs2292832, and rs11614913 polymorphisms and risk of CRC in overall studies or in different ethnic groups for all genetic models. But significant association between rs2910164 and CRC was observed in hospital based studies in the recessive model. And we found that rs2292832 polymorphisms may be associated with increased the risk of CRC in population based studies for recessive model. While T allele of rs11614913 was a protect factor of CRC in the homozygous model when we excluded the studies which were not in agreement with HWE.

Although the biological mechanism of SNPs in miRNAs contributing to the regulation of cancer susceptibility and development remains unknown, a large quantity of meta-analyses has been engaged in the newly developed field in the past few years [23]. A previous meta-analysis by K. Srivastava and A. Srivastava [23] was demonstrated that miR-196a2 rs11614913 polymorphisms have significant associations with overall cancer risk. In Xu et al.'s meta-analysis [24], TT genotype of rs11614913 polymorphism was associated with decreased cancer risk. Rs2910164 C allele was associated with decreased overall cancer risk especially for cervical cancer and prostate cancer risk in Chinese population. Rs3746444 G allele was a risk factor in Chinese population, especially for breast cancer. Different results were also presented in several meta-analyses which evaluated the relationship between miRNAs and CRC risk [25]. Wan et al.'s study demonstrates that miR-196a2 rs11614913 most likely contributes to decreased risk of CRC, whereas miR-146a rs2910164 may not be associated with the susceptibility to CRC [25]. While the pooled data from Du et al.'s meta-analysis supported that the miR-196a2 rs11614913 and miR-149 rs2292832 polymorphisms may contribute to susceptibility to CRC [26]. The latest meta-analysis, which related to the associations between miRNA polymorphisms and colorectal cancer, performed by Wu et al. indicated that SNP rs11614913 but not SNP rs2910164 and SNP rs2292832 may contribute to susceptibility to CRC in an Asian-specific manner [27]. This result was unconformable with our results. Compared with Wu's meta-analysis which included 9 relevant studies, our research totally identified 15 studies including 5,486 CRC cases and 7,184 controls. Thus, our meta-analysis contained the newest data and largest sample size on the study of the relationship between miRNAs and colorectal cancer. Additionally, we performed subgroup analyses by different data information in more detail.

Some limitations still existed in this meta-analysis. Firstly, the included studies are mainly based on Asian background. There were only five studies based on Caucasian background and no studies on African background. Secondly, some detailed information (such as sex, age, life-style, and environmental factors) was not considered. Further large scale multicenter studies based on Caucasian or African will be needed to clarify the possible roles of these polymorphisms in CRC. Though many investigators have been devoted to identify the roles of the miRNAs in carcinogenesis, the mechanisms were still unclear. Further biologically functional studies are warranted to explain the molecular mechanisms.

In summary, this meta-analysis showed that there were no associations between the four polymorphisms and CRC risk in the overall population. Further analysis stratified by the source of control showed that rs2910164 polymorphism is associated with CRC risk in hospital based studies, and rs2292832 polymorphism may contribute to the susceptibility of CRC in population based studies. In the pooled analysis from all studies which are in agreement with HWE revealed that rs11614913 TT carriers may have a decreased CRC risk. Moreover, in the stratified analysis according to ethnicity, rs3746444 polymorphism may reduce the risk of CRC in Caucasians.

## Supplementary Material

Table S1: Meta-analysis results.

## Figures and Tables

**Table 1:** Characteristics of the studies included in the meta-analysis.

First author	Year	Country	Ethnicity	Genotyping method	Source of control	Number (case/control)	SNP number
Dikaiakos [28]	2015	Greek	Caucasian	PCR-RFLP	HB	157/299	1, 3, 4
Kupcinskas [29]	2014	Lithuania, Latvia	Caucasian	TaqMan	HB	193/428	1, 3
Mao [30]	2014	China	Asian	SNPScan	PB	554/566	1
Hu [31]	2014	China	Asian	PCR-RFLP	HB	276/373	1, 4
Wu [15]	2014	China	Asian	ASA	HB	175/300	1, 2, 4
Lv [8]	2013	China	Asian	PCR-RFLP	PB	353/540	1, 2, 3, 4
Chae [21]	2013	Korea	Asian	PCR-RFLP	PB	399/568	1
Ma [32]	2013	China	Asian	TaqMan	PB	1147/1203	1
Vinci [16]	2013	Italy	Caucasian	TaqMan	HB	160/178	1, 2, 3, 4
Zhang [17]	2012	China	Asian	PCR-RFLP	PB	478/477	2, 3
Hezova [20]	2012	Czech	Caucasian	TaqMan	HB	197/212	1, 3
Min [22]	2012	Korea	Asian	PCR-RFLP	PB	446/502	1, 2, 3, 4
Zhu [33]	2012	China	Asian	TaqMan	PB	573/588	3
Chen [34]	2012	China	Asian	PCR-LDR	HB	126/407	3
Zhan [35]	2011	China	Asian	PCR-RFLP	PB	252/543	3

HWE: Hardy-Weinberg equilibrium; PB: population based; HB: hospital based; PCR: polymerase chain reaction; RFLP: restriction fragment length polymorphism; LDR: ligation detection reaction; ASA: allele-specific amplification; SNP: single-nucleotide polymorphisms; SNP number 1: miR-146-a G>C (rs2910164); 2: miR-149 C>T (rs2292832); 3: miR-196a-2 C>T (rs11614913); 4: miR-499 A>G (rs3746444).

**Table 2:** MiRNA polymorphisms genotype distribution and allele frequency in cases and controls.

First author	Genotype (*N*)								Allele frequency (*N*)					
	Case				Control				Case		Control		MAF	HWE (*P* value)
	Total	AA	Aa	aa	Total	AA	Aa	aa	A	a	A	a		
rs2910164														
Dikaiakos 2015 [28]	157	8	48	101	299	21	120	158	64	250	162	436	0.80	0.782
Kupcinskas 2014 [29]	193	140	50	2	428	275	134	15	330	54	684	164	0.14	0.789
Mao 2014 [30]	554	70	291	186	566	85	271	205	431	663	441	681	0.61	0.768
Hu 2014 [31]	200	34	82	84	373	44	187	142	250	150	275	471	0.38	0.137
Wu 2014 [15]	175	22	59	80	300	53	120	114	103	219	226	348	0.68	**0.035**
Lv 2013 [8]	331	54	230	47	513	96	274	143	338	324	560	466	0.49	0.080
Chae 2013 [21]	399	61	182	156	568	121	282	165	304	494	524	612	0.62	0.980
Ma 2013 [36]	1147	444	534	169	1203	397	614	192	1422	872	1408	998	0.38	0.075
Vinci 2013 [16]	160	17	57	86	178	13	65	100	91	229	91	265	0.72	0.590
Hezova 2012 [20]	197	115	70	12	212	124	79	9	300	94	327	97	0.24	0.415
Min 2012 [22]	446	151	233	62	502	188	245	69	535	357	621	383	0.40	0.443

rs2292832														
Wu 2014 [15]	175	21	123	28	300	76	58	116	165	179	210	290	0.52	**<0.001**
Lv 2013 [8]	347	30	64	253	459	48	103	308	124	570	199	719	0.82	**<0.001**
Vinci 2013 [16]	160	79	58	23	178	86	75	17	216	104	247	109	0.33	0.912
Zhang 2012 [14]	443	50	190	203	435	46	202	187	290	596	294	576	0.67	0.431
Min 2012 [22]	446	48	177	221	502	51	219	232	273	619	321	683	0.69	0.948

rs11614913														
Dikaiakos 2015 [28]	157	19	69	69	299	33	149	117	107	207	215	383	0.66	0.156
Kupcinskas 2014 [29]	193	79	87	27	428	199	174	54	245	141	572	282	0.37	0.104
Lv 2013 [8]	374	10	223	114	531	109	331	91	243	451	549	413	0.60	**<0.001**
Vinci 2013 [16]	160	62	86	12	178	83	84	11	210	110	250	106	0.34	0.087
Hezova 2012 [20]	197	82	89	26	212	87	103	22	253	141	277	147	0.36	0.291
Zhang 2012 [14]	455	79	204	172	463	81	197	185	362	548	359	567	0.60	**0.026**
Min 2012 [22]	466	120	201	125	502	100	254	148	441	451	454	550	0.48	0.633
Zhu 2012 [33]	573	140	303	130	588	121	295	172	583	563	537	639	0.49	0.790
Chen 2012 [34]	126	27	64	35	407	94	206	107	118	134	394	420	0.53	0.788
Zhan 2011 [35]	252	68	128	56	543	112	267	163	264	240	493	593	0.48	0.890

rs3746444														
Dikaiakos 2015 [28]	157	85	64	8	299	182	99	18	234	80	463	135	0.25	0.361
Hu 2014 [31]	211	157	49	5	373	282	81	10	363	59	654	101	0.14	0.162
Wu 2014 [15]	175	96	17	38	300	141	44	63	209	93	326	170	0.31	**<0.001**
Lv 2013 [8]	346	258	88		504	366	138						—	—
Vinci 2013 [16]	160	35	32	93	178	17	56	105	102	218	90	266	0.68	**0.026**
Min 2012 [22]	446	292	142	12	502	334	154	14	726	166	822	182	0.19	0.453

A: the major allele; a: the minor allele; MAF: minor allele frequencies.
