# Supplementary material for: Quantitative Assessment of the Association between Genetic Variants in MicroRNAs and Colorectal Cancer Risk
Source: Biomed Res Int. 2015 May 20;2015:276410. doi: 10.1155/2015/276410 (PMC4452836; doi:10.1155/2015/276410)
Supplement: Supplementary file 1 — Table S1: Meta-analysis results. [file 276410.f1.docx]

**Table S1** Meta-analysis results

| Comparisons | | Studies | OR | 95%CI | *P* value | Heterogeneity | | Effects model |
| --- | --- | --- | --- | --- | --- | --- | --- | --- |
|  |  | Case/Control |  |  |  | I^2^ | *P* value |  |
| rs2910164 | | | | | | | | |
| Total studies | | 11( 3,937/5,120) | | | | | | |
| C vs. G | | 0.96  1.02  1.00  1.03 | | 0.82–1.12 | 0.62 | 82% | <0.00001 | R |
| CC vs. GG | |  |  | 0.78–1.33 | 0.89 | 67% | 0.0008 | R |
| GC vs. GG | |  |  | 0.83–1.20 | 0.97 | 60% | 0.005 | R |
| CC vs. GC+GG | |  |  | 0.80–1.31 | 0.83 | 79% | <0.00001 | R |
| CC+GC vs. GG | | 1.01 | | 0.84–1.22 | 0.89 | 63% | 0.003 | R |
| Studies which agreement with HWE | | 10(3,776/4,833) | | | | | | |
| C vs. G | | 0.93 | | 0.79–1.09 | 0.38 | 82% | <0.00001 | R |
| CC vs. GG | | 0.97 | | 0.73–1.28 | 0.83 | 67% | 0.001 | R |
| GC vs. GG | | 0.98 | | 0.81–1.20 | 0.88 | 64% | 0.003 | R |
| CC vs. GC+GG | | 0.99 | | 0.76–1.27 | 0.91 | 79% | <0.00001 | R |
| CC+GC vs. GG | | 0.99 | | 0.82–1.20 | 0.90 | 64% | 0.003 | R |
| Asian | | 7(3,231/4,007) | | | | | | |
| C vs. G | | 1.03 | | 0.88–1.21 | 0.71 | 81% | <0.0001 | R |
| CC vs. GG | | 1.04 | | 0.77–1.41 | 0.80 | 75% | 0.0005 | R |
| GC vs. GG | | 1.07 | | 0.83–1.37 | 0.60 | 73% | 0.001 | R |
| CC vs. GC+GG | | 1.00 | | 0.75–1.33 | 0.99 | 84% | <0.00001 | R |
| CC+GC vs. GG | | 1.08 | | 0.86–1.37 | 0.51 | 73% | 0.001 | R |
| Caucasian | | 4(706/1,113) | | | | | | |
| C vs. G | | 0.82 | | 0.53–1.26 | 0.36 | 85% | 0.0001 | R |
| CC vs. GG | | 0.93 | | 0.60–1.43 | 0.73 | 52% | 0.10 | F |
| GC vs. GG | | 0.83 | | 0.64–1.06 | 0.14 | 0% | 0.71 | F |
| CC vs. GC+GG | | 1.17 | | 0.89–1.52 | 0.26 | 60% | 0.06 | F |
| CC+GC vs. GG | | 0.84 | | 0.66–1.07 | 0.16 | 20% | 0.29 | F |
| PB | | 5(2,870/3,347) | | | | | | |
| C vs. G | | 1.00 | | 0.83–1.20 | 0.97 | 84% | <0.0001 | R |
| CC vs. GG | | 1.02 | | 0.71–1.46 | 0.93 | 80% | 0.0005 | R |
| GC vs. GG | | 1.15 | | 0.87–1.52 | 0.32 | 77% | 0.002 | R |
| CC vs. GC+GG | | 0.90 | | 0.63–1.29 | 0.56 | 88% | <0.00001 | R |
| CC+GC vs. GG | | 1.12 | | 0.86–1.46 | 0.39 | 77% | 0.002 | R |
| HB | | 6(1,067/1,773) | | | | | | |
| C vs. G | | 0.96 | | 0.68–1.26 | 0.61 | 84% | <0.0001 | R |
| CC vs. GG | | 1.02 | | 0.77–1.36 | 0.87 | 52% | 0.06 | F |
| GC vs. GG | | 0.82 | | 0.66–1.01 | 0.06 | 0% | 0.43 | F |
| CC vs. GC+GG | | 1.24 | | 1.03–1.49 | **0.02** | 42% | 0.13 | F |
| CC+GC vs. GG | | 0.87 | | 0.71–1.06 | 0.18 | 41% | 0.13 | F |
| rs2292832 | | | | | | | | |
| Total studies | | 5(1,568/1,824) | | | | | | |
| T vs. C | | 1.05  1.09  1.35  0.91 | | 0.95–1.17 | 0.36 | 39% | 0.16 | F |
| TT vs. CC | |  |  | 0.87–1.37 | 0.44 | 0% | 0.73 | F |
| CT vs. CC | |  |  | 0.64–2.86 | 0.43 | 92% | <0.00001 | R |
| TT vs. CT+CC | |  |  | 0.54–1.53 | 0.71 | 91% | <0.00001 | R |
| TT+CT vs. CC | | 1.24 | | 0.82–1.87 | 0.31 | 76% | 0.002 | R |
| Studies which agreement with HWE | | 3(1,049/1,115) | | | | | | |
| T vs. C | | 1.06 | | 0.94–1.21 | 0.35 | 0% | 0.98 | F |
| TT vs. CC | | 1.07 | | 0.81–1.42 | 0.63 | 0% | 0.62 | F |
| CT vs. CC | | 0.86 | | 0.66–1.11 | 0.24 | 0% | 1.00 | F |
| TT vs. CT+CC | | 1.16 | | 0.97–1.39 | 0.10 | 0% | 0.63 | F |
| TT+CT vs. CC | | 0.94 | | 0.74–1.20 | 0.63 | 0% | 0.99 | F |
| Asian | | 4(1,408/1,646) | | | | | | |
| T vs. C | | 1.05  1.05  1.53 | | 0.94–1.17 | 0.43 | 54% | 0.09 | F |
| TT vs. CC | |  |  | 0.83–1.34 | 0.67 | 0% | 0.75 | F |
| CT vs. CC | |  |  | 0.59–3.97 | 0.39 | 93% | <0.00001 | R |
| TT vs. CT+CC | | 0.81 | | 0.45–1.46 | 0.48 | 93% | <0.00001 | R |
| TT+CT vs. CC | | 1.33 | | 0.79–2.24 | 0.28 | 80% | 0.002 | R |
| PB | 3(1,236/1,396) | | | | | | | |
| T vs. C | | 1.11 | | 0.98–1.25 | 0.11 | 0% | 0.44 | F |
| TT vs. CC | | 1.09 | | 0.84–1.41 | 0.53 | 0% | 0.66 | F |
| CT vs. CC | | 0.89 | | 0.68–1.17 | 0.41 | 0% | 0.91 | F |
| TT vs. CT+CC | | 1.18 | | 1.01–1.38 | **0.04** | 0% | 0.70 | F |
| TT+CT vs. CC | | 1.01 | | 0.79–1.30 | 0.94 | 0% | 0.62 | F |
| HB | 2(332/428) | | | | | | | |
| T vs. C | | 0.90 | | 0.73–1.11 | 0.34 | 56% | 0.13 | F |
| TT vs. CC | | 1.11 | | 0.69–1.77 | 0.66 | 15% | 0.28 | F |
| CT vs. CC | | 2.52 | | 0.29–22.05 | 0.40 | 97% | <0.00001 | R |
| TT vs. CT+CC | | 0.59 | | 0.09–4.01 | 0.59 | 95% | <0.00001 | R |
| TT+CT vs. CC | | 1.72 | | 0.54–5.50 | 0.36 | 91% | 0.0006 | R |
| rs11614913 | | | | | | | | |
| Total studies | | 10(2,906/4,150) | | | | | | |
| T vs. C | | 1.10 | | 0.84–1.43 | 0.50 | 93% | <0.00001 | R |
| TT vs. CC | | 1.18 | | 0.76–1.83 | 0.47 | 87% | <0.00001 | R |
| CT vs. CC | | 1.11 | | 0.82–1.50 | 0.49 | 82% | <0.00001 | R |
| TT vs. CT+CC | | 1.07 | | 0.82–1.38 | 0.63 | 79% | <0.00001 | R |
| TT+CT vs. CC | | 1.13 | | 0.83–1.55 | 0.44 | 85% | <0.00001 | R |
| Studies which agreement with HWE | | 8(2,104/3,156) | | | | | | |
| T vs. C | | 0.96 | | 0.84–1.09 | 0.52 | 57% | 0.02 | R |
| TT vs. CC | | 0.80 | | 0.68–0.95 | **0.009** | 39% | 0.12 | F |
| CT vs. CC | | 0.91 | | 0.79–1.04 | 0.17 | 42% | 0.10 | F |
| TT vs. CT+CC | | 0.89 | | 0.78–1.02 | 0.10 | 40% | 0.11 | F |
| TT+CT vs. CC | | 0.92 | | 0.76–1.12 | 0.42 | 50% | 0.05 | R |
| Asian | | 6(2,199/3,034) | | | | | | |
| T vs. C | | 1.07 | | 0.72–1.61 | 0.73 | 96% | <0.00001 | R |
| TT vs. CC | | 1.19 | | 0.62–2.29 | 0.60 | 93% | <0.00001 | R |
| CT vs. CC | | 1.16 | | 0.72–1.87 | 0.54 | 89% | <0.00001 | R |
| TT vs. CT+CC | | 1.00 | | 0.70–1.44 | 0.99 | 88% | <0.00001 | R |
| TT+CT vs. CC | | 1.17 | | 0.71–1.91 | 0.54 | 91% | <0.00001 | R |
| Caucasian | | 4(707/1,116) | | | | | | |
| T vs. C | | 1.13 | | 0.98–1.30 | 0.09 | 0% | 0.88 | F |
| TT vs. CC | | 1.16 | | 0.84–1.60 | 0.37 | 0% | 0.92 | F |
| CT vs. CC | | 1.12 | | 0.90–1.39 | 0.33 | 4% | 0.37 | F |
| TT vs. CT+CC | | 1.21 | | 0.93–1.57 | 0.15 | 0% | 0.98 | F |
| TT+CT vs. CC | | 1.15 | | 0.93–1.42 | 0.19 | 0% | 0.52 | F |
| PB | | 5(2,073/2,627) | | | | | | |
| T vs. C | | 1.08 | | 0.67–1.72 | 0.76 | 97% | <0.00001 | R |
| TT vs. CC | | 1.21 | | 0.57–2.59 | 0.62 | 94% | <0.00001 | R |
| CT vs. CC | | 1.19 | | 0.68–2.08 | 0.54 | 91% | <0.00001 | R |
| TT vs. CT+CC | | 0.99 | | 0.65–1.50 | 0.96 | 90% | <0.00001 | R |
| TT+CT vs. CC | | 1.19 | | 0.67–2.11 | 0.55 | 92% | <0.00001 | R |
| HB | | 5(833/1,523) | | | | | | |
| T vs. C | | 1.12 | | 0.98–1.27 | 0.09 | 0% | 0.94 | F |
| TT vs. CC | | 1.15 | | 0.87–1.53 | 0.32 | 0% | 0.97 | F |
| CT vs. CC | | 1.11 | | 0.91–1.36 | 0.31 | 0% | 0.53 | F |
| TT vs. CT+CC | | 1.18 | | 0.94–1.47 | 0.16 | 0% | 0.99 | F |
| TT+CT vs. CC | | 1.14 | | 0.94–1.38 | 0.17 | 0% | 0.68 | F |
| rs3746444 | | | | | | | | |
| Total studies | | 6(1,471/2,104) | | | | | | |
| G vs. A | | 0.96 | | 0.84–1.10 | 0.58 | 26% | 0.25 | F |
| GG vs. AA | | 0.90 | | 0.67–1.22 | 0.50 | 470% | 0.11 | F |
| AG vs. AA | | 0.83 | | 0.54–1.26 | 0.38 | 78% | 0.001 | R |
| GG vs. AG+AA | | 0.95 | | 0.73–1.25 | 0.73 | 0% | 1.00 | F |
| GG+AG vs. AA | | 0.91 | | 0.71–1.17 | 0.45 | 62% | 0.02 | R |
| Studies which agreement with HWE | | 3(814/1,174) | | | | | | |
| G vs. A | | 1.07 | | 0.91–1.26 | 0.42 | 0% | 0.80 | F |
| GG vs. AA | | 0.95 | | 0.57–1.59 | 0.85 | 0% | 0.99 | F |
| AG vs. AA | | 1.13 | | 0.93–1.38 | 0.22 | 0% | 0.54 | F |
| GG vs. AG+AA | | 0.90 | | 0.54–1.49 | 0.68 | 0% | 0.97 | F |
| GG+AG vs. AA | | 1.11 | | 0.92–1.35 | 0.28 | 0% | 0.62 | F |
| Asian | | 4(1,154/1,627) | | | | | | |
| G vs. A | | 0.98 | | 0.83–1.15 | 0.80 | 0% | 0.58 | F |
| GG vs. AA | | 1.17 | | 0.80–1.71 | 0.41 | 0% | 0.72 | F |
| AG vs. AA | | 0.98 | | 0.79–1.22 | 0.87 | 43% | 0.17 | F |
| GG vs. AG+AA | | 0.97 | | 0.67–1.41 | 0.87 | 0% | 0.98 | F |
| GG+AG vs. AA | | 0.96 | | 0.81–1.13 | 0.60 | 0% | 0.55 | F |
| Caucasian | | 2(317/477) | | | | | | |
| G vs. A | | 0.92 | | 0.58–1.48 | 0.74 | 76% | 0.04 | R |
| GG vs. AA | | 0.57 | | 0.34–0.95 | **0.03** | 52% | 0.15 | F |
| AG vs. AA | | 0.64 | | 0.13–3.08 | 0.58 | 93% | 0.0001 | R |
| GG vs. AG+AA | | 0.94 | | 0.64–1.38 | 0.74 | 0% | 0.77 | F |
| GG+AG vs. AA | | 0.72 | | 0.21–2.46 | 0.60 | 91% | 0.0009 | R |
| PB | | 2(792/1,006) | | | | | | |
| G vs. A | | 1.03 | | 0.82–1.30 | 0.79 | - | - | F |
| GG vs. AA | | 0.98 | | 0.45–2.15 | 0.96 | - | - | F |
| AG vs. AA | | 1.05 | | 0.80–1.39 | 0.71 | - | - | F |
| GG vs. AG+AA | | 0.96 | | 0.44–2.11 | 0.93 | - | - | F |
| GG+AG vs. AA | | 0.98 | | 0.80–1.21 | 0.88 | 0% | 0.48 | F |
| HB | | 4(679/1,098) | | | | | | |
| G vs. A | | 0.93 | | 0.79–1.10 | 0.39 | 39% | 0.18 | F |
| GG vs. AA | | 0.89 | | 0.64–1.23 | 0.47 | 60% | 0.06 | F |
| AG vs. AA | | 0.74 | | 0.40–1.37 | 0.34 | 83% | 0.0006 | R |
| GG vs. AG+AA | | 0.95 | | 0.71–1.27 | 0.73 | 0% | 0.99 | F |
| GG+AG vs. AA | | 0.83 | | 0.53–1.31 | 0.43 | 76% | 0.006 |  |

F: fixed effects model; R: random effects model.
